# Automatic extraction of protein-protein interactions using grammatical relationship graph

**DOI:** 10.1186/s12911-018-0628-4

**Published:** 2018-07-23

**Authors:** Kaixian Yu, Pei-Yau Lung, Tingting Zhao, Peixiang Zhao, Yan-Yuan Tseng, Jinfeng Zhang

**Affiliations:** 1 Department of Statistics, Florida State University, Tallahassee, FL 32306 USA; 2 Department of Biostatistics, University of Texas MD Anderson Cancer Center, Houston, TX 77054 USA; 3 Department of Geography, Florida State University, Tallahassee, FL 32306 USA; 4 Department of Computer Science, Florida State University, Tallahassee, FL 32306 USA; 5 Center for Molecular Medicine and Genetics, School of Medicine, Wayne State University, Detroit, MI 48201 USA

**Keywords:** Information extraction, Relationship extraction, Protein-protein-interactions, Nature language processing, Graph-theoretic algorithm

## Abstract

**Background:**

Relationships between bio-entities (genes, proteins, diseases, etc.) constitute a significant part of our knowledge. Most of this information is documented as unstructured text in different forms, such as books, articles and on-line pages. Automatic extraction of such information and storing it in structured form could help researchers more easily access such information and also make it possible to incorporate it in advanced integrative analysis. In this study, we developed a novel approach to extract bio-entity relationships information using Nature Language Processing (NLP) and a graph-theoretic algorithm.

**Methods:**

Our method, called GRGT (Grammatical Relationship Graph for Triplets), not only extracts the pairs of terms that have certain relationships, but also extracts the type of relationship (the word describing the relationships). In addition, the directionality of the relationship can also be extracted. Our method is based on the assumption that a triplet exists for a pair of interactions. A triplet is defined as two terms (entities) and an interaction word describing the relationship of the two terms in a sentence. We first use a sentence parsing tool to obtain the sentence structure represented as a dependency graph where words are nodes and edges are typed dependencies. The shortest paths among the pairs of words in the triplet are then extracted, which form the basis for our information extraction method. Flexible pattern matching scheme was then used to match a triplet graph with unknown relationship to those triplet graphs with labels (True or False) in the database.

**Results:**

We applied the method on three benchmark datasets to extract the protein-protein-interactions (PPIs), and obtained better precision than the top performing methods in literature.

**Conclusions:**

We have developed a method to extract the protein-protein interactions from biomedical literature. PPIs extracted by our method have higher precision among other methods, suggesting that our method can be used to effectively extract PPIs and deposit them into databases. Beyond extracting PPIs, our method could be easily extended to extracting relationship information between other bio-entities.

## Background

Relationships among different biological terms such as genes, proteins, diseases, small molecules, pathways, and gene ontology (TO) terms (called bio-entities in this paper) form the backbone of our knowledge. Bio-entity relationships such as protein-protein interactions (PPIs) are indispensable for understanding of complex diseases, biological processes, and guiding drug discoveries [1]. Human annotation has been used in the past to extract this information from scientific literature, which is then deposited into various databases [2–21].

However, human annotation can be very time and resource consuming, and keeping pace with the ever increasing amount of biomedical publications has become more and more difficult. As a result, computational methods have been designed to extract bio-entity relationships automatically from the literature, and used to assist scientists in their efforts to build databases using manual annotation approach [22–48]. Most computational studies attempted to extract PPIs from PubMed abstracts due to the easy accessibility of deposited articles [49, 50]. Most of the PPI extraction methods are based on one of the two ways: (1) specify some rules (or patterns, templates etc.) manually [34, 50–66]; or (2) infer/learn the rules computationally from manually labeled sentences [67–69].

Simple rules, such as co-occurrence, were used in the early efforts of PPI extraction. Co-occurrence assumes that two proteins likely interact with each other if they co-occurred in the same sentence/abstract [70, 71]. The drawback of these approaches is that the false positive rate of the methods tends to be quite high. Later studies used manually-specified rules, which can sometimes achieve much lower false positive rate, but often suffered from low recall rate [34, 50–66].

Recently, machine learning solutions have been proposed to extract PPI information automatically. By learning the language rules from annotated texts, machine learning techniques can perform better than other methods in terms of both decreasing the false-positive rate and increasing the coverage [67–69]. Huang et al.*...* [67] used a dynamic programming algorithm, similar to that used for sequence alignment, to extract patterns from sentences tagged by part-of-speech taggers. Kim et al.*.* [69] and Murugesan et al [72] used a kernel-based approach for learning genetic and protein-protein interaction patterns.

Although extensive studies have by far been carried out, existing methods only achieved partial success in small datasets [55, 58–60, 67, 73] [54]. Kim et al [74] developed a web server: PIE, and tested their method on BioCreative dataset [38, 39, 75], achieving a reasonably good performance for a PPI article filtering task.

A machine learning based PPI extraction method was developed by Chowdhary et al. [73]. In this study, a novel methodology was developed based on Bayesian networks (BNs) for extracting PPI triplets (a PPI triplet consists of two protein names and the corresponding interaction word) from unstructured text. Various of features were extracted from sentences with potential PPIs, including preposition close to the protein names, the preposition close to the interaction word, the type of interaction word, the order of the words in the triplet, the distance between the first and second triplet word, the distance betwenn the second and third triplets words, existence of comma between triplet words, the distance of the comma to one of the triplet word, existence of the negative words such as “but”, “not”, “no” etc., existence of “which”, and number of interaction words in the sentence, in addition to other features. The method achieved an overall accuracy of 87% on a cross-validation test using manually annotated dataset with 2550 triplets. It was also showed, through extracting PPI triplets from a large number of PubMed abstracts, that the method was able to complement human annotations to extract large number of new PPIs from literature. Through manual validation of some of the predictions, they concluded that the current databases likely missed at least 130,000 PPIs [45]. The method was later applied to a large scale PPI extraction task for automatic knowledge discovery using an integrated bio-entity network made using heterogeneous types of bio-entities, including proteins, genes, diseases, gene oncology terms, pathways etc. [45]. A variation of the method that allows the extraction of directionality was also developed later using a mixture logistic model and ensemble approach [76]. A new PPI corpus, called PICAD (Protein Interaction Corpus with Annotated Directions), was manually curated with more than 1500 sentences and more than 10,000 triplet cases.

Thus far, there have been few methods that extract both the protein names and the interaction words at the same time. However, only the protein names are insufficient to understand PPIs. As a result, there is an urgent need to extract the PPI triplet (two different protein names and one interact word) in order to reveal how the proteins are interacted [77].

**Fig. 1:**
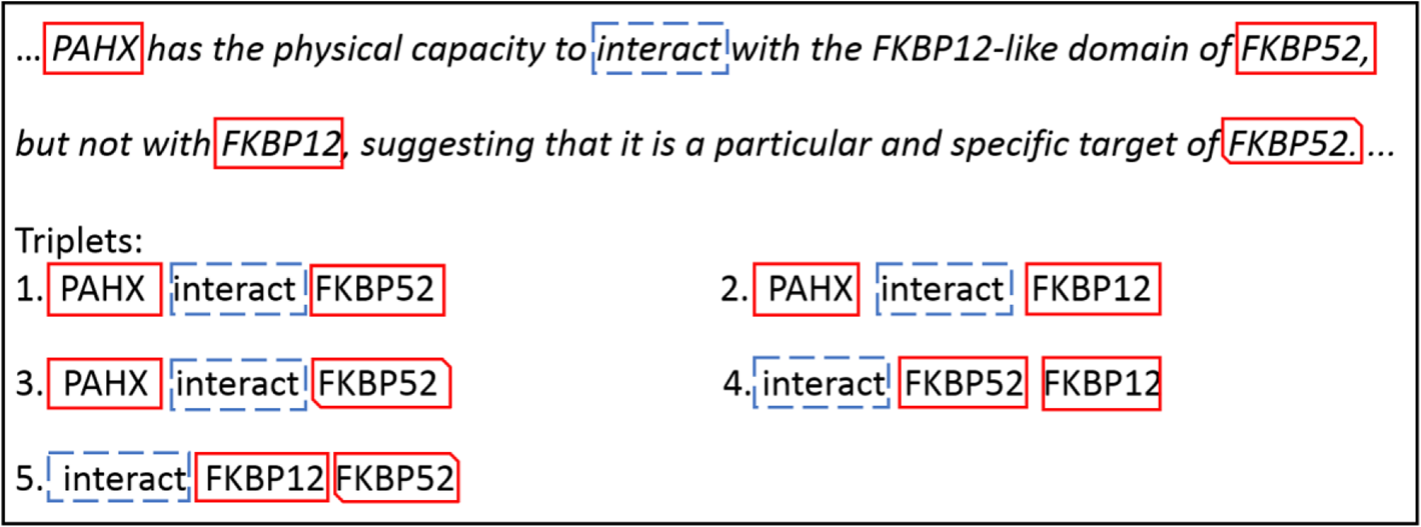
Example of PPIs. The sentence has four protein names and two interactions words, “interact” and “target”. The five triplets with “interact” are shown below the sentence

There is a practical issue in extracting PPI triplets if we omit the structure of a sentence. Ideally the PPI triplet appears in the order of (protein1---interaction word --- protein2), and one single sentence contains only one triplet; In practice, however, a PPI triplet ordered as (interaction word --- protein1 --- protein2) may occur, and for each sentence, multiple distinguished triplets may exist as well. In most cases, there is only one triplet that describes the true PPI. For example, the sentence in Fig. 1 contains four protein names (FKBP12-like is not considered as a protein name) PAHX, FKBP52, FKBP12, and FKBP52 (the second occurrence of FKBP52 in the sentence) and one interaction word *interacts*. There are five PPI triplets (Fig. 1), only one of the triplets correctly describes this specific PPI (triplet 1 in Fig. 1).

Recently Natural Language Processing (NLP) techniques have been utilized in many machine learning approaches [63–66] to parse sentences into dependency trees or constituent trees, which could further be used in pattern matching or rule-based search. However, to our best knowledge, all the methods have to adopt some given rules/patterns. The given rules are typically rather general; therefore, they fail to represent all the patterns in the training sentences.

Bui et al. has developed a hybrid approach for extracting PPIs [78]. The method consists of two phases. First, the data were automatically categorized into subsets based on its semantic properties and candidate PPI pairs were extracted from these subsets. Second, support vector machines (SVMs) were applied to classify candidate PPI pairs using features specific for each subset. They obtained promising results on five benchmark datasets: AIMed, BioInfer, HPRD50, IEPA and LLL with F-scores ranging from 60 to 84%.

A comprehensive benchmark was developed for Kernel based PPI extraction methods by Tikk et al. [43]. In the work, the authors study whether the reported performance metrics are robust across different corpora and learning settings and whether the use of deep parsing actually leads to an increase in extraction quality. They concluded that for most kernels no sensible estimation of PPI extraction performance on new text is possible, given the current heterogeneity in evaluation data [43].

In this paper, we propose a method based on NLP and automatically learn rules/patterns to extract the PPI triplets from sentences. We then classify them as true or false with probabilities based on whether the interaction words correctly describe the interaction relationship between the two participant protein names.

## Methods

Our method, GRGT, utilized the grammatical relationship among each Protein-Protein-Interaction triplet extracted by natural language processing (NLP) techniques and a graph theorem algorithm (shortest path algorithm) as feature to build a classifier. A dictionary of protein names and interaction words with their morphemes were built based on our previous study [28]. All interaction words in our dictionary were a single word, and were grouped manually into 22 categories by the similarity of their grammatical properties to reflect the fact that some interaction word can be used interchangeably without altering the sematic of the sentence.

### Preprocessing

**Fig. 2:**
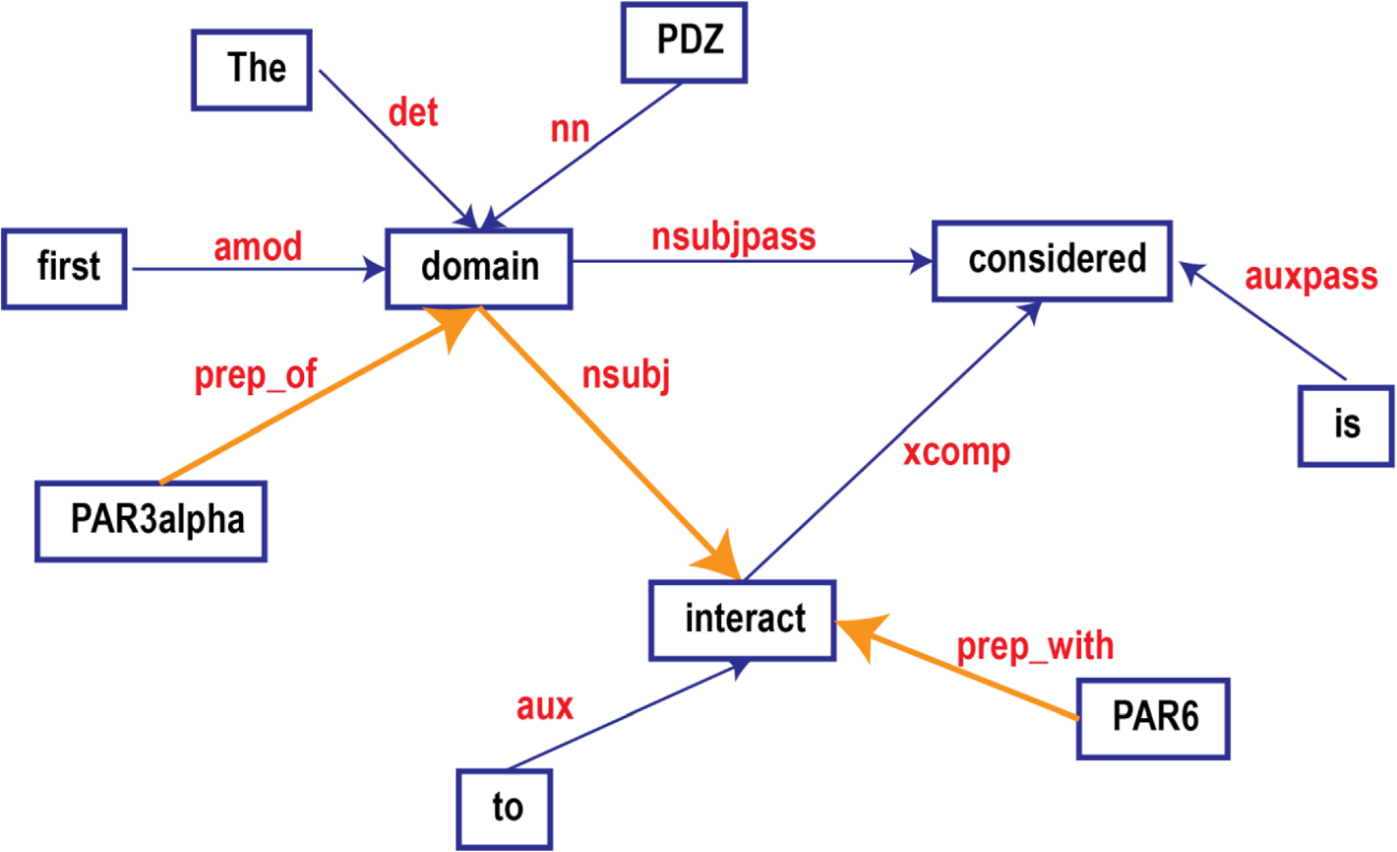
Grammatical dependencies graph

The sentence harboring the PPI triple was first tokenized, so that each word took their own tag as an independent component. The tokenized sentence was then parsed using Stanford Sentence Parser to obtain the grammatical relationships among all the words. For example, the sentence, *“The first PDZ domain of PAR3alpha is considered to interact with PAR6.,”* was parsed to have a relationship graph showing in Fig. 2 representing the grammatical relationships between the words in the sentence. The words in red, such as *nn, nsubj*, etc. are typed dependencies defined in [79].

The typed dependencies have a hierarchical structure themselves. Here we only introduce some necessary facts. The top level of the hierarchical structure is dependent (*dep*), which has the following types: auxiliary (*aux*), argument (*arg*), coordination (*cc*), conjunct (*conj*), expletive (*expl*), modifier (*mod*), parataxis (*parataxis*), punctuation (*punct*), referent (*ref*) and semantic dependent (*sdep*). Each of the above types may have subtypes themselves. For example, *arg* has subtypes: agent (agent), complement (*comp*) and subject (*subj*), where *subj* has nominal subject (*nsubj*) and clausal subject (*csubj*) as its subtypes. For example, “domain” is *nsubj* of “interact” (Fig. 2).

### Feature extraction

**Fig. 3:**
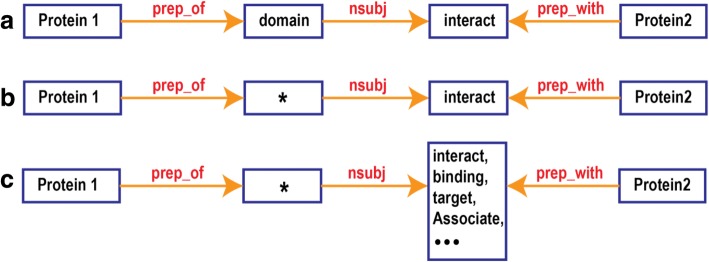
Grammatical dependencies sub-graph: **a**) the strict pattern directly extracted from the annotated sample; **b**) the relaxed pattern where the word "domain" was allowed to vary; **c**) the more general pattern where the interaction word can be replaced by the ones from the same pre-defined interaction class

We designed the direct feature of each triplet (two protein names and the interaction word) as the minimal sub-graph containing the triplet. Dijkstra’s shortest paths algorithm was adopted to find the shortest path (highlighted path in Fig. 2) in the grammatical graph between pairs of the triplet elements. The obtained sub-graph is the Grammatical Relationship Graph for Triplets (GRGT) (Fig. 3a).

The GRGT of Fig. 3a describes the meaning *“domain of P1 (PAR3alpha) interact with P2 (PAR6).”* The information in this graph is all the information we need to know to infer the interaction between PAR3alpha and PAR6. In fact, for two triplets with only altered protein names but exactly the same GRGT, these two triples are equivalent in the sense of grammatical relations; thus, they shall be classified as the same category. Although the direct feature, exact GRGT, is quite specific and the classification based on only these exact GRGTs are of very high precision. It sacrifices the generalizability a lot: the pattern of a new GRGT of PPI triplet has to match the training true patterns exactly to be considered as a true PPI. To introduce more general GRGTs, we could relax the subgraph. For example, in the subgraph above, we allowed the *domain* to vary from annotated samples (Fig. 3b). Furthermore, it is also possible to alternating the interaction word to replace the interaction word in GRGT with other members in its group (notice we grouped interaction words into 22 categories).

### Training

We adopted a probabilistic way to train the model. Each feature, GRGT, will be assigned a probability of being corresponding to a true PPI as the proportion of true PPI triplets in the training data having such a feature (either a direct or generalized one) in all triplets that have this feature.

The directions of the sub-graph can also be inferred at the same time, since the information of the direction of the true patterns can also be annotated.

### Prediction

**Fig. 4:**
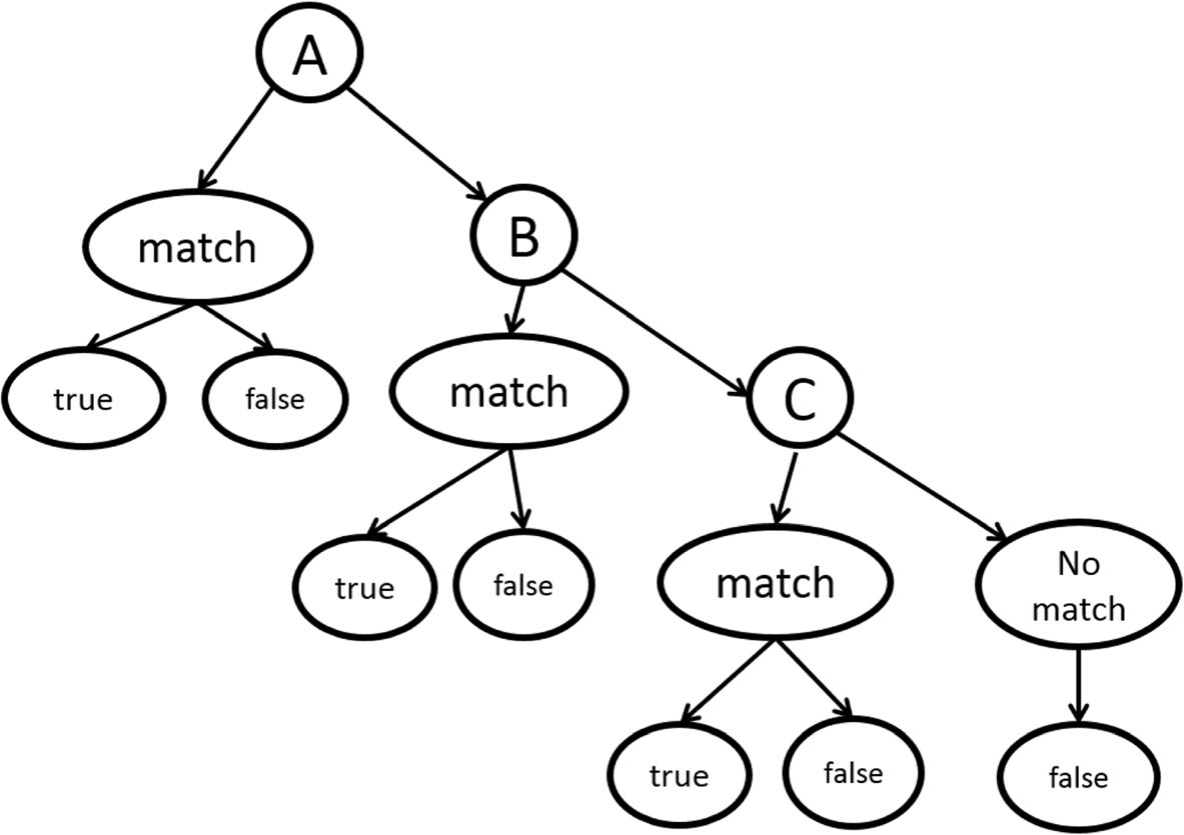
Example decision tree

A simple decision tree (Fig. 4) was used to cast the prediction. The decision tree has one decision node at each level representing the GRGT at different levels of generalizations. For simplicity, we use the above interaction sentence *“domain of P1 interact with P2”* as the annotated training sample to demonstrate how the decision tree works. The procedure is shown below:

- The first level of the decision tree will be the exact feature in Fig. 3a. If the new sentence does not match the pattern exactly, send this sentence to the second level. Therefore, *“domain of P1 interact with P2”* is a match, and the probability of triplet *“P1-interact-P2”* being true is assigned as the probability of this feature being associated with true PPIs. However, *“motif of P1 interact with P2”* does not match the feature, thus should be passed to the next level.
- The second level is the relaxed graph as shown in Fig. 3b. At this level, the previous example, *“motif of P1 interact with P2”* is a match; therefore, the probability of this triplet *P1-interact-P2* classified as true triplet is the associated probability of the feature. However, the sentence *“motif of P1 associates with P2”* does not have a feature in this level since the interaction word is different. Therefore, it is passed to the next level.
- The third level, as described in Fig. 3c, is the most relaxed version. In this level we allow the interaction words to differ from the annotated example as long as they belong to the same group. For example, the above sentence *“motif of P1 associates with P2”* is a match in level 3, although it is not a match in level 1 or 2. Therefore the triplet *P1-associates-P2* is given the probability being true as the probability of the feature being true. If a sentence fails to match the pattern in this level (in practice there may be much more levels), we mark the triplet contained in this sentence as a false triplet.

## Results

Table 1 summarizes the datasets we used for testing the performance of our method (GRGT), including three benchmark datasets: HPRD50, IEPA, LLL, and a corpus we constructed: PICAD (protein interaction corpus with annotated directions). PICAD contains not only the interactions of protein pairs, but the directionality of interactions, which is important for analyzing biological network.

**Table 1:** Dataset information

Corpus	No. of sentences	No. of Triplets	No. of true PPI
HPRD50	145	954	126
IEPA	374	1341	164
LLL	79	977	106
PICAD	1033	19,755	1831

**Fig. 5:**
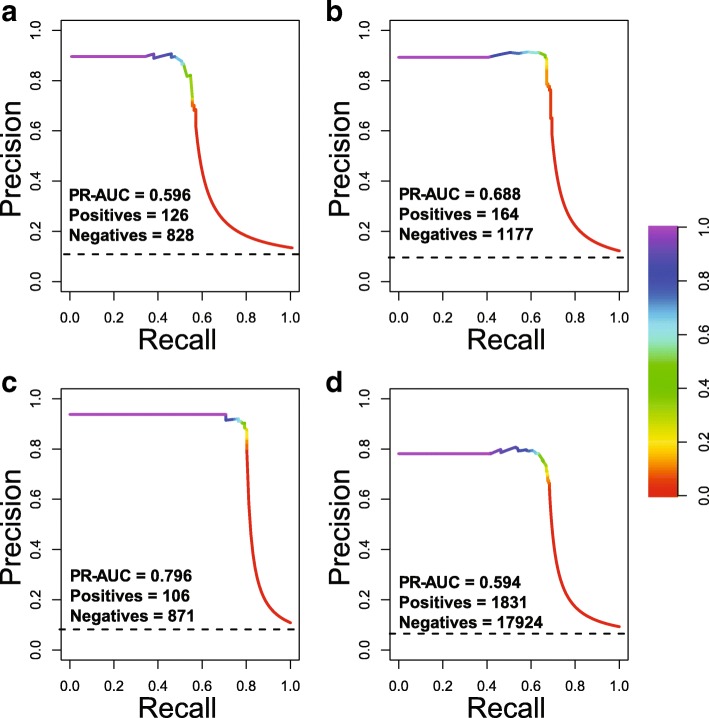
Precision-recall curve: **a**) HPRD50, **b**) IEPA, **c**) LLL, **d**) PICAD

Table 2 shows the experiment results based on leave-one-out classification. The performance of top-performing methods in literature [23, 47, 48, 72] was also included for comparison. Compared with other methods, GRGT largely improved precision while maintaining comparable F-score, especially on IEPA and LLL. High precision is very important when the discovered (classified) results are going to be used as prior knowledge to guide experiment design. If one model has low precision, the results could be doubtful, and the researchers would receive incorrect information, which may provide false guidance for downstream studies. On the other hand, the lower recall rate of GRGT resulted from that most misclassified cases were false negatives, where true triplets cannot be matched to any known patterns. This would be acceptable since most true interactions (PPI triplets) tend to occur more than once in literature. The interaction will be extracted as long as one of PPI triplets is classified as true. A system with high precision can thus be used to more effectively extract PPIs from biomedical literature and deposit them into databases. In such task, the value of precision would be more important than the value of recall, and the tradeoff that decreases F-score with improving precision significantly is worthy. Figure 5 shows the precision-recall curves of our system on different datasets.

**Table 2:** Performance comparison

Corpus	HPRD50			IEPA			LLL			PICAD^b^		
	F	P	R	F	P	R	F	P	R	F	P	R
Bui et al. [24]	71.7	62.2	84.7	73.4	62.9	88.1	83.6	81.9	85.4	–	–	–
Miwa et al. [49]	70.9	68.5	76.1	71.7	67.5	78.6	80.1	77.6	86.0	–	–	–
Chang et al. [48]	71.5	63.8	81.2	71.4	62.5	83.3	80.6	73.2	89.6	–	–	–
Murugesan et al. [73]	80.0	76.3	84.2	80.2	75.9	85.2	89.2	87.3	91.2	–	–	–
^a^Zhao et al. [81]	71.3	58.7	92.4	74.2	67.0	84.0	82.0	75.8	91.8	–	–	–
GRGT	64.0	86.5	50.8	74.9	91.0	63.6	83.6	91.2	77.1	70.0	78.2	63.4

Performance comparison of our method (GRGT) with top-performing methods on four benchmark datasets. *F* F_1_-score, *P* precision, *R* recall. The measurement is out of 100. ^a^deep learning method. ^b^Values are not available because of the unavailability of executable program or source code

Recently, several studies introduced deep learning methods for PPI extraction [80–83]. We also compare the performance of our method with Zhao et al. [83], which uses the same benchmark datasets. Our method again had better precision for the benchmark datasets compared with [83], while they get improvement in recall.

## Discussion

To further improve the performance of GRGT, the patterns can be simplified further so that more true triplets can be matched if they are similar to true patterns, but not exactly the same. The hierarchical structure of the typed dependencies can be used for this purpose. For example, *nsubj* (nominal subject) can be reduced to *subj* (subject) or even further to *arg* (argument). We need to balance recall and precision rate while doing this, as simplification would improve the recall rate, but with a cost of lowered precision rate. Some more experiments can be performed on various ways of reducing the exact patterns, and on how to combine the new relaxed patterns with our existing patterns by designing different decision trees to achieve better performance.

We further analyzed the extracted patterns (subgraphs), and in Table 3 we can see that not a lot of patterns appeared more than once, only about 10–20% of the extracted subgraphs appeared at least twice in the entire dataset, which leaves the coverage of triplets per sample relatively low, so that there is not much information can be borrowed from other triplets in the dataset. To further improve the performance, one can annotate more interaction cases to increase the size of the training set, which should significantly improve the recall rate of our method since we will have more coverage per pattern.

**Table 3:** Summary of the extracted subgraphs and their generalizations

Corpus	# of patterns	# of valid patterns^a^	Triplet per valid pattern
HPRD50	3895	522	1.83
IEPA	6117	575	2.33
LLL	4859	891	1.10
PICAD	18,794	4363	4.53

^a^Patterns appeared at least twice

This method can be used to extract other relationships as well, as long as the triplet is well defined and the library for terms and interaction words are given.

Consistent with literature, Table 2 showed that deep learning approaches cannot beat traditional kernel-based or machine-learning methods all the time in PPI extraction task. The reasons would be 1) deep neural networks would not be beneficial without effectively large amount of training data and 2) deep neural networks are relatively difficult to train because of the large number of parameters. For a well-trained deep neural networks from large amount of training data, the performance may still get improved by combining with traditional feature-based machine learning methods [84]. The features we designed in this study can be applied to other machine learning methods, as well as be incorporated into deep learning methods. The current work is an expanded version of a previous study [82].

## Conclusions

In this work, we developed a new NLP-based method, GRGT, for extracting the protein-protein interactions from biomedical literature. The performance of GRGT was demonstrated by comparing with top performing methods using benchmark datasets. GRGT obtained better precision, indicating that researchers can use PPIs extracted by GRGT as prior knowledge to guide experiment design with high confidence. We believe that GRGT will be a very useful tool for PPI-extraction task.

## Abbreviations

GRGT: Grammatical Relationship Graph for Triplets
NLP: Natural language processing
PICAD: Protein interaction corpus with annotated directions
PPI: Protein-protein interaction
